# The Effect of Automated Mammogram Orders Paired With Electronic Invitations to Self-schedule on Mammogram Scheduling Outcomes: Observational Cohort Comparison

**DOI:** 10.2196/27072

**Published:** 2021-12-07

**Authors:** Frederick North, Elissa M Nelson, Rebecca J Buss, Rebecca J Majerus, Matthew C Thompson, Brian A Crum

**Affiliations:** 1 Division of Community Internal Medicine Department of Internal Medicine Mayo Clinic Rochester, MN United States; 2 Enterprise Office of Access Management Mayo Clinic Rochester, MN United States; 3 Department of Neurology Mayo Clinic Rochester, MN United States

**Keywords:** electronic health record, schedule, patient appointment, preventive health service, office visit, outpatient care, mammogram, software tool, computer software application, mobile applications, self-schedule, app, EHR, screening, diagnostic, cancer

## Abstract

**Background:**

Screening mammography is recommended for the early detection of breast cancer. The processes for ordering screening mammography often rely on a health care provider order and a scheduler to arrange the time and location of breast imaging. Self-scheduling after automated ordering of screening mammograms may offer a more efficient and convenient way to schedule screening mammograms.

**Objective:**

The aim of this study was to determine the use, outcomes, and efficiency of an automated mammogram ordering and invitation process paired with self-scheduling.

**Methods:**

We examined appointment data from 12 months of scheduled mammogram appointments, starting in September 2019 when a web and mobile app self-scheduling process for screening mammograms was made available for the Mayo Clinic primary care practice. Patients registered to the Mayo Clinic Patient Online Services could view the schedules and book their mammogram appointment via the web or a mobile app. Self-scheduling required no telephone calls or staff appointment schedulers. We examined uptake (count and percentage of patients utilizing self-scheduling), number of appointment actions taken by self-schedulers and by those using staff schedulers, no-show outcomes, scheduling efficiency, and weekend and after-hours use of self-scheduling.

**Results:**

For patients who were registered to patient online services and had screening mammogram appointment activity, 15.3% (14,387/93,901) used the web or mobile app to do either some mammogram self-scheduling or self-cancelling appointment actions. Approximately 24.4% (3285/13,454) of self-scheduling occurred after normal business hours/on weekends. Approximately 9.3% (8736/93,901) of the patients used self-scheduling/cancelling exclusively. For self-scheduled mammograms, there were 5.7% (536/9433) no-shows compared to 4.6% (3590/77,531) no-shows in staff-scheduled mammograms (unadjusted odds ratio 1.24, 95% CI 1.13-1.36; *P*<.001). The odds ratio of no-shows for self-scheduled mammograms to staff-scheduled mammograms decreased to 1.12 (95% CI 1.02-1.23; *P*=.02) when adjusted for age, race, and ethnicity. On average, since there were only 0.197 staff-scheduler actions for each finalized self-scheduled appointment, staff schedulers were rarely used to redo or “clean up” self-scheduled appointments. Exclusively self-scheduled appointments were significantly more efficient than staff-scheduled appointments. Self-schedulers experienced a single appointment step process (one and done) for 93.5% (7553/8079) of their finalized appointments; only 74.5% (52,804/70,839) of staff-scheduled finalized appointments had a similar one-step appointment process (*P*<.001). For staff-scheduled appointments, 25.5% (18,035/70,839) of the finalized appointments took multiple appointment steps. For finalized appointments that were exclusively self-scheduled, only 6.5% (526/8079) took multiple appointment steps. The staff-scheduled to self-scheduled odds ratio of taking multiple steps for a finalized screening mammogram appointment was 4.9 (95% CI 4.48-5.37; *P*<.001).

**Conclusions:**

Screening mammograms can be efficiently self-scheduled but may be associated with a slight increase in no-shows. Self-scheduling can decrease staff scheduler work and can be convenient for patients who want to manage their appointment scheduling activity after business hours or on weekends.

## Introduction

About 1 in 8 women in the United States will develop breast cancer during her life [1]. Breast cancer screening with mammograms can help detect breast cancer at an early stage when treatment is most successful [2]. Despite the need for breast cancer screening, 31% of women in the screening age range of 45-55 years have not had a mammogram in the last 2 years [3]. Several interventions have been tried to increase the percentage of women receiving screening mammograms [4]. Primary care health care providers have historically played a major role in advising patients about screening mammography. Typically, providers address preventive health services, including screening mammography, during the periodic examination [5]. However, despite some continued promotion of the periodic health care examination [6], there is no overwhelming evidence for the periodic examination to significantly change health outcomes, including breast cancer [7]. In addition, screening mammography is often just one of many recommended actions that primary care providers need to address with their patients. In a study at Mayo Clinic, we found that primary care patients aged 50-65 years, on average, had 5.5 unmet health care recommendations, with the conclusion that there needs to be “new approaches to address the burgeoning numbers of uncompleted recommendations” [8]. Yarnall et al [9] also noted the large amount of time that is required for primary care providers to address every preventive service, including screening mammography.

Automated ordering and self-scheduling of screening mammography with the assistance of the electronic health record (EHR) is an intervention that could help deliver the preventive service of early breast cancer detection in a primary care practice. Criteria for screening mammography can often be found within the EHR. For example, the American Cancer Society recommends mammograms up to every year for women aged 40-75 years depending on the life expectancy [2]. Determining whether a screening mammogram is due for a given individual can be accomplished through software rules that query the EHR for patient characteristics and dates of previous mammograms. Self-scheduling has been used for airline, hotel, and event bookings for years. So why has the self-scheduling of medical appointments lagged? The short answer is that medical appointments encompass many different appointment types and appointment purposes that require very different rules for scheduling. For example, Zocdoc.com is an internet third party medical appointment enabler that matches individuals on the web with health care providers for scheduled visits. Zocdoc makes some of the details of the matching process available [10,11]. Scheduling in Zocdoc includes very specific rules such as matching insurance coverage, preferred medical specialty, and availability for face-to-face or video visit [12,13]. COVID-19 visits are another very specific visit type requiring specific criteria for booking. In a recent study, Judson et al [14] noted how self-triage rules in the self-scheduling process were designed to limit COVID video visits to those who did not require more emergent care. Because of the differences in appointment purpose and type, the COVID-19 self-scheduling rules are very different from those used by Zocdoc for more general appointments. The periodic well-child examination is another example of a self-scheduling appointment type that requires a completely different set of rules. Scheduling of the well-child examination is based on the age of the child, the date of the last well-child examination, and matching with the child’s primary care provider [15].

The screening mammogram appointment is also a visit type with its own unique set of rules that distinguish it from other visit types. The unique challenges for self-scheduling screening mammograms are (1) there are specific criteria for patient age, date of the last mammogram, and whether a screening mammogram is appropriate; (2) it is a radiologic procedure requiring an electronic order; (3) there are patient and provider requirements so that the assignment and communication of results is assured. In addition to examining the outcomes of self-scheduled mammograms, we show our automated processes for the self-scheduled screening mammogram visit that address the unique challenges of this visit type.

## Methods

### Setting

The implementation of automated ordering paired with self-scheduling of mammograms took place at Mayo Clinic in 2019. Mayo Clinic is a multispecialty group practice with several locations in the United States and internationally. Our study focuses on the screening mammogram process of the primary care practices of Mayo Clinic for 12 consecutive months from September 2019 through August 2020. Mayo Clinic has primary care practices in the United States in Florida, Arizona, and many locations in the upper Midwest, primarily in the states of Minnesota, Wisconsin, and Iowa. All the primary care sites were included in this study. This study was limited to the bilateral breast screening mammogram examination, which is the recommended radiographic procedure for the early detection of breast cancer.

### Automated Screening Mammogram Order Process

**Figure 1:**
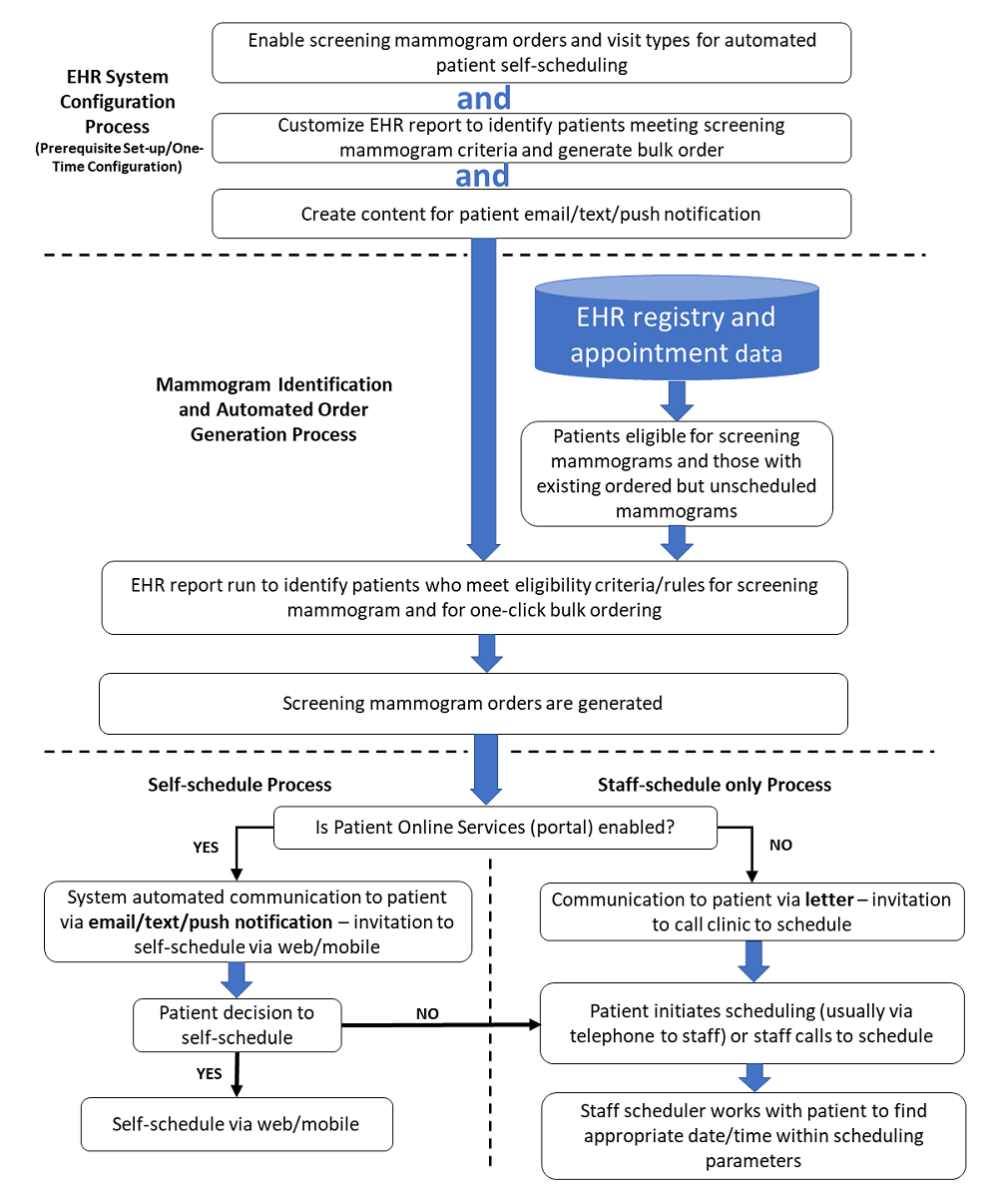
Prerequisite system configuration and process flow for automated identification of eligible patients for screening mammograms, automated mammogram order generation, and communication to patients for self-scheduling versus staff scheduling. EHR: electronic health record.

At Mayo Clinic, screening mammography requires an order for the specific imaging examination. Patients are not allowed to self-order a mammogram. However, Mayo Clinic has developed a process with rules that automatically generates orders for screening mammograms, allowing one-click bulk ordering of hundreds of mammograms by a single provider. The top part of Figure 1 shows the one-time EHR system configuration set-up needed for the mammogram bulk ordering process. The configuration of the order and visit types in the scheduling system were needed to allow automated mammogram ordering. A special EHR report was configured to identify patients meeting the screening mammogram criteria and to produce the bulk mammogram order. Creation of patient email/text/push notification content was also required for the self-schedule electronic invitation process.

After the prerequisite EHR system configurations are completed, the mammogram ordering process starts by using EHR data to identify patients who are eligible and due for screening mammography. The appointment scheduling system is also queried for those who are due and have a mammogram ordered but not scheduled. For those who do not have an active mammogram order but are due for a mammogram, a mammogram order is created. Thus, all patients who are due for a mammogram either have a mammogram order generated automatically to enable scheduling or they are identified to enable scheduling if an active mammogram order is already in place but not yet scheduled. Figure 1 (bottom third) shows that once the mammogram order is generated or identified as needing scheduling, the process diverges depending on whether the patient is enabled with patient online services. All patients who are due and had the mammogram order generated or who have an existing mammogram order needing scheduling are sent invitations to schedule their mammograms. Those who use patient online services are sent invitations by an email message and, if mobile app, a push notification. Those without patient online services are sent a letter by post. The mammogram invitations sent by the portal included an invitation to self-schedule. All those with patient online services are enabled to self-schedule both by using web and mobile app. Patients with patient online services also have the option to have staff help them schedule their mammograms (staff scheduled) via a phone call or portal message. For patients without patient online services, mammograms can only be scheduled with staff assistance (staff scheduler).

### Staff Scheduling Versus Self-scheduling

Staff schedulers are clinic staff employees who schedule or cancel appointments for patients. Until the self-scheduling process was implemented, staff employee appointment schedulers were responsible for working with patients and radiology schedules to schedule mammograms. Patients, whether patient online services–enabled or not, can schedule their mammograms by telephone or in person via staff appointment schedulers. Appointment scheduling via staff schedulers normally occurs during business hours of 7 AM to 5 PM on weekdays. Appointment schedulers have the ability to schedule mammograms more than 12 weeks into the future. Self-scheduling via patient online services can be done either via web or via mobile and is available 24/7. Patients can directly see the mammogram scheduling template for the days that they select and can click on the appointment time that they want. Self-schedulers are restricted to scheduling their mammogram in a 12-week rolling window from the day that they could schedule. Self-schedulers are also not allowed to double book.

### Appointment Definitions

Self-schedulers or self-cancelers are the patients who used the Mayo software interface (web or mobile) to self-schedule or self-cancel the mammogram appointments. It should be noted that we focus on self-schedule actions in this study. There were some patients who never used the self-scheduling feature but self-cancelled the appointments made by the staff schedulers. To be considered self-scheduled, a patient had to have at least one appointment action of self-scheduling (booking an appointment with the self-schedule software). The few patients who self-cancelled their staff-scheduled appointment were classified as staff-scheduled.

**Figure 2:**
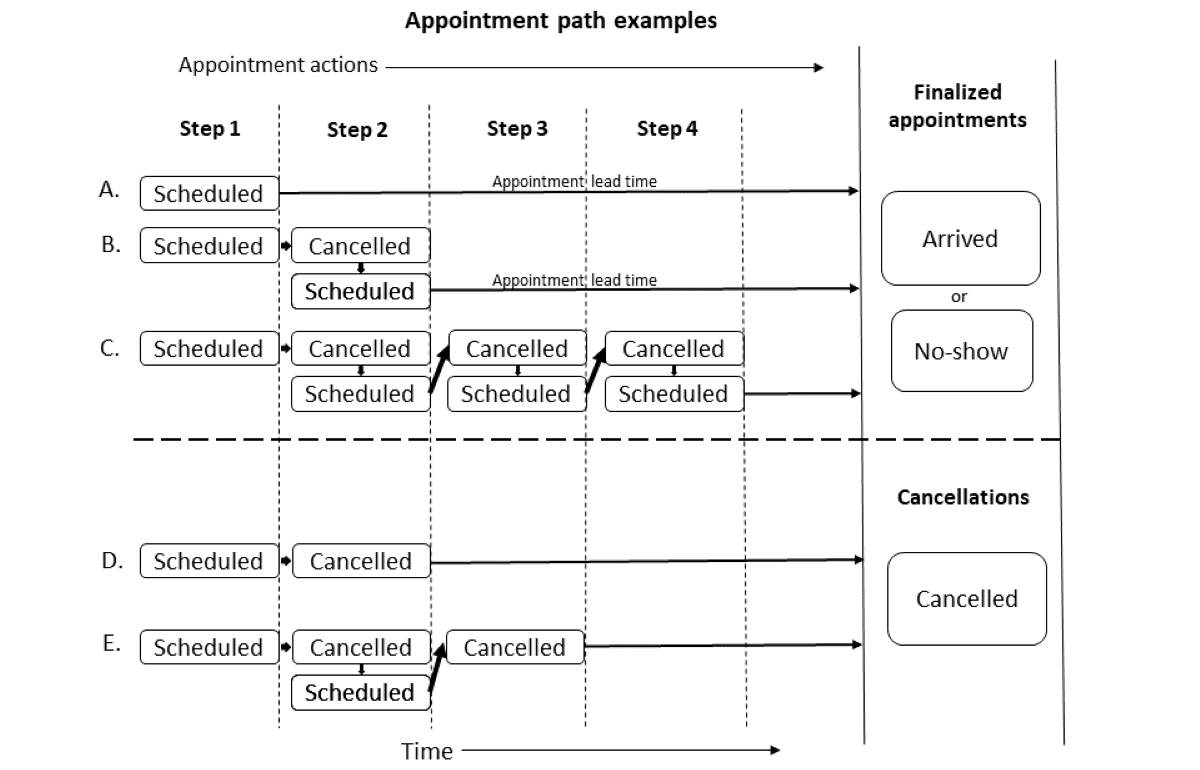
Examples of different appointment paths showing the appointment actions and appointment steps leading to a finalized appointment or cancellation.

An appointment action is either a schedule or cancel event. With the self-scheduling process, appointment actions could be done either by the patient (self) or staff. An appointment path is the sequence of appointment actions leading to a finalized appointment or cancellation outcome (Figure 2). Appointment paths can contain both self and staff appointment actions. The example above of a self-cancelled appointment that was scheduled by a staff would have 2 appointment actions: a staff-scheduler action and a self-cancel action.

Finalized appointments were those scheduled appointments that were left scheduled up to the appointment date and time (not cancelled before appointment time). Figure 2 shows examples of appointment paths and appointment outcomes. Our data start with a time-stamped appointment schedule action. We dichotomized appointment actions into those by staff schedulers and those by self-schedulers. As shown in Figure 2, each patient (whether self-scheduled or staff-scheduled) begins with a scheduling action that we term as appointment step 1. Patients can then go through several decision steps of whether to cancel or reschedule (a cancel and schedule pair). Some patients would reschedule multiple times before a finalized appointment. To quantify this activity, we counted the appointment steps. Figure 2A shows an appointment path to appointment finalization with just 1 step, the initial scheduling action. Figure 2B and Figure 2C show appointment paths to appointment finalization taking 2 and 4 steps, respectively. Appointment paths ending in a cancellation outcome also may take several appointment steps. Figure 2D and Figure 2E show cancellation examples that take 2 and 3 appointment steps, respectively, to result in a cancellation.

Appointment outcomes are dichotomously categorized as finalized appointments or cancellations. Finalized appointments are further dichotomously categorized as completed or no-shows (never arrived at the scheduled appointment time). Figure 2A also shows the appointment lead time, which is the scheduled appointment date/time minus the date/time the appointment was made. This is the lead time that the patient has from the date of scheduling the appointment to the actual future-reserved appointment date.

### Mammogram Appointment Selection and Follow-up

Our data source was EHR-generated scheduling and cancelling information on all bilateral screening mammogram appointments made for primary care patients for the 12 months from September 1, 2019 through August 31, 2020. Scheduled mammogram appointments were either cancelled or completed (patient arrived or no-show) by September 1, 2020 for our follow-up on finalized appointments and no-shows. Patients eligible for automated ordering and invitation to schedule a mammogram were primary care practice patients of Mayo Clinic in Arizona or Florida or in the Mayo Clinic Health System (Minnesota, Wisconsin, Iowa). Self-scheduling through web or mobile required patient online service registration; staff scheduling was available for all patients who had an active mammogram order, regardless of patient online service registration status.

### Summary of the Outcome Measures

A finalized scheduled mammogram was the outcome of scheduling and cancelling actions as shown in Figure 2. A finalized mammogram appointment was defined as a mammogram appointment scheduled and remaining active until the date and time of the scheduled mammogram radiology visit. Scheduling and cancelling actions were outcomes of interest defined as the scheduling of a mammogram (booking an assigned time and date for the mammogram) or the action of cancelling a mammogram (cancelling a previously booked mammogram appointment). Scheduling and cancelling actions were dichotomized depending on whether they were accomplished by self-scheduling or by staff schedulers. The no-show mammogram appointment, defined as the finalized appointment for a patient who never arrived for their scheduled mammogram, was also an outcome of interest. Appointment lead time was defined as the time difference between the actual appointment date and time and the date and time it was last scheduled, after any prior schedule and cancel actions as noted in Figure 2. Appointment lead times were of interest because staff schedulers could schedule mammogram appointments beyond the 12-week lead time limit of self-scheduling. Patient uptake of the self-scheduling process was measured as counts and percentage of patients over time who used self-scheduling or a combination of self-scheduling and self-cancelling exclusively or in combination with staff scheduling for their appointment actions for a finalized appointment. The mutually exclusive 3 categories of patients who finalized appointments were as follows: self-scheduled exclusively (could also self-cancel), self- and staff-scheduled (any combination of self and staff scheduling actions), and patients who used staff schedulers exclusively (no self-scheduling or self-cancelling appointment actions).

### Data Collection and Analysis

Data were collected by the Epic EHR of Mayo Clinic. Patients who either staff-scheduled or self-scheduled were registered patients of Mayo Clinic. In addition, we limited this study to portal-registered patients who were established primary care patients; therefore, there were essentially complete demographic data available for each patient (age, race, sex, ethnicity). Any uncategorized or missing information on race or ethnicity was placed in the other or unknown category. Appointment data were entered by the Epic scheduling software. Dates and times of self-scheduling and staff scheduling were automatically entered into the EHR software by patient record number and categorized on data entry as being sourced from self-scheduling (patient online services) or by the staff scheduler. Mammograms were not done unless the patient was checked in by radiology staff as “arrived.” If the patient did not arrive and the radiology staff overlooked listing the patient as a no-show, an EHR scheduling rule marked the visit as a no-show 72 hours after the scheduled appointment to ensure the capture of these overlooked no-shows.

We categorized scheduling and cancelling actions according to whether they occurred outside of the usual business hours (Monday through Friday, 7 AM to 5 PM). The proportion of mammogram appointment lead times over 12 weeks was calculated for both staff-scheduled and self-scheduled appointments. As mentioned above, only those scheduled for mammography who were registered with patient online services were analyzed. Thus, portal registration status was not in our primary analysis. Those without portal registration were included in additional analysis as described below. Age is a known confounder for no-shows in radiology visits [16]; therefore, we adjusted for age in our analysis of no-shows. We conducted additional analyses to determine how sensitive our findings were to the disruption that the COVID-19 pandemic had on mammogram appointments. In March 2020, shortly after the midpoint of our data capture, mammogram appointments were suspended temporarily. It was unclear how much this disruption of scheduling affected self-scheduling activity and whether it increased the use of staff schedulers. To quantify this, we analyzed separately the 6 pre-COVID months and the 6 post-COVID months (September 2019 through February 2020 and March 2020 through August 2020, respectively) for self-scheduling and staff-scheduling activity. We also performed additional data analysis to evaluate the self-scheduling uptake for all patients scheduling mammograms, including those without portal registration.

### Statistical Analysis

We used JMP 14.3 (SAS Institute Inc) for the statistical analysis. The chi-square test was used for categorical analysis. We used logistic regression analysis in a model to explain the differences in the no-shows adjusted by patient age to control for age as a known confounder in radiology no-shows [16]. A logistic regression analysis model using age, race, and ethnicity was also used to adjust for additional differences in demographics for the no-shows analysis.

### Ethics

This was a retrospective study examining quality measures and uptake of a self-scheduling process. Self-scheduling was a voluntary additional option offered to all primary care patients with patient online services; all individuals could continue to schedule their mammograms with staff schedulers if that was their preference (see patient decision point in Figure 1). This study met the institutional review board criteria for exemption (IRB-2020-006809).

## Results

### Uptake of Self-scheduling Mammograms

**Figure 3:**
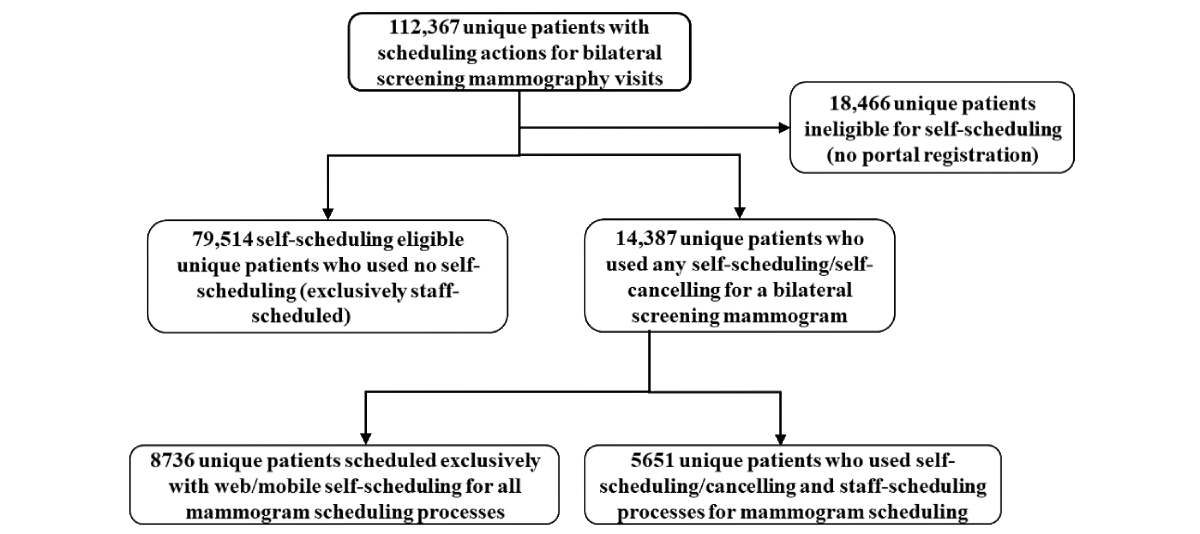
Patients who had scheduling actions for bilateral screening mammograms for the 12 months of the study. Patient counts show those who exclusively used self-scheduling, those exclusively staff-scheduled, and those who had both self-scheduling and staff-scheduling appointment actions.

Figure 3 shows the patient counts of those who had mammograms scheduled for the 12 months of the study. Approximately 16.4% (18,466/112,367) of the patients did not have access to either self-scheduling or self-cancelling (not registered with patient online services). In this study, we focused on 93,901 individuals who had access to self-scheduling. Of those individuals, 15.3% (14,387/93,901) used self-scheduling or self-cancelling. Of those with patient online services, 9.3% (8736/93,901) exclusively used self-scheduling and thus did not use any staff-scheduler resources. Another 6% (5651/93,901) used some self-scheduling/self-cancelling processes to arrange their screening mammogram.

**Figure 4:**
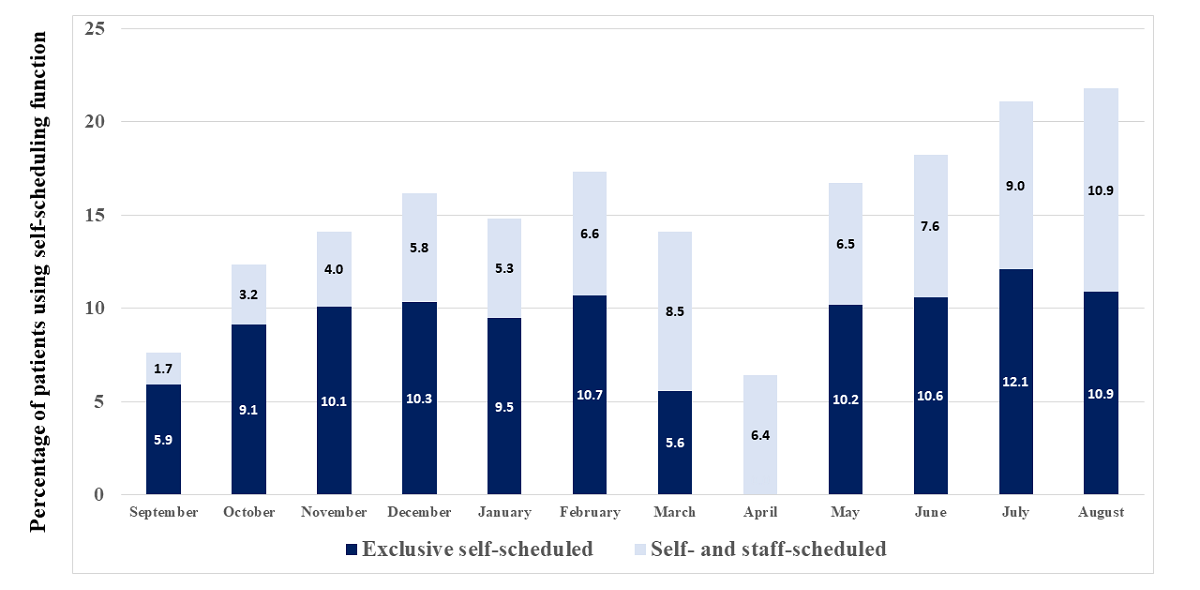
Longitudinal uptake of self-scheduling paired with automatically generated invitations to schedule mammograms (September 2019 to August 2020). The graph shows the percentage of patients with patient online services–enabled who either exclusively used self-scheduling or used some self-scheduling. Self-cancelling activity took place in April 2020 when patients could not self-schedule.

Figure 4 shows the longitudinal percentage uptake of self-scheduling for those who had self-scheduling access. In the initial month of widespread implementation, 7.6% (678/8898) of all individuals involved in scheduling mammograms were doing some self-scheduling actions. Eleven months later (July 2020), this had increased by 276%, so that 21.1% (1991/9442) of the patients scheduling mammograms were doing some self-scheduling. At 12 months (August 2020), 21.8% (1091/5005) of the patients were doing some self-scheduling, but since many patients who started scheduling in August had not reached the scheduled date of their appointment (finalized their appointment) by the end of data collection (August 31, 2020), the counts were lower. The drop in scheduling in March and April 2020 was associated with access limitations imposed during the initial months of the COVID-19 pandemic. As part of those restrictions, self-scheduling was not available for scheduling mammograms during part of March 2020 and all of April 2020, but self-cancelling was still available.

### Demographics of the Patients

Table 1 compares the demographics of the individuals who had patient online services and performed any self-scheduling activity with those of individuals who had staff-scheduled appointments. There were notable differences in the age distributions, which was consistent with younger individuals being more comfortable with web and mobile technology. Although statistically there were some racial differences, the absolute percentages were similar. The percentage of White females in the self-scheduled was 93.7% (13,474/14,382) compared to 93.7% (74,436/79,476) in the staff scheduled, thereby showing a nonsignificant difference (*P*=.90).

**Table 1:** Demographics of individuals who used self-scheduling compared to those of individuals who used staff-scheduling for making appointments for their screening mammograms.

Demographic characteristic		Any self-scheduled, (n=14,387), n (%)	Exclusively staff-scheduled (n=79,514), n (%)	*P* value^a^	
**Age (years)**					<.001
	20-29	2 (0.01)	40 (0.05)		
	30-39	91 (0.63)	606 (0.76)		
	40-49	4311 (29.96)	15,113 (19.01)		
	50-59	4468 (31.06)	21,322 (26.82)		
	60-69	3954 (27.48)	24,977 (31.41)		
	70-79	1408 (9.79)	14,675 (18.46)		
	80-89	148 (1.03)	2674 (3.36)		
	90-99	5 (0.03)	107 (0.13)		
Self-described gender (female)		14,382 (99.97)	79,476 (99.95)	.50	
**Race**					.002
	White	13,474 (93.65)	74,436 (93.61)		
	Black	186 (1.29)	1357 (1.71)		
	Asian	316 (2.20)	1577 (1.98)		
	Other	269 (1.87)	1420 (1.79)		
	Not disclosed	142 (0.99)	724 (0.91)		
**Ethnicity**					<.001
	Hispanic	336 (2.34)	2339 (2.94)		
	Not Hispanic	13,772 (95.73)	75,745 (95.26)		
	Undisclosed/unknown	279 (1.94)	1430 (1.80)		

^a^Null hypothesis (H0) tested: percentage of each demographic characteristic is equal between those who performed any self-scheduled activity and those who had staff-scheduled appointments exclusively.

### Appointment Actions Completed by Self-schedulers

As mentioned in Methods, before an appointment is finalized, it can be cancelled and rescheduled many times. Table 2 shows the counts of all the scheduling and cancelling appointment actions done by self-scheduled patients and those done by staff schedulers. Out of 175,256 appointment actions completed, 10% (17,475/175,256) were done by patients. All the appointment actions resulted in a total of 86,964 finalized appointments, with 10.8% (9433/86,964) at least partially finalized by the patient.

**Table 2:** Appointment metric comparison between self-scheduled and staff-scheduled appointments for those with access to self-scheduling (patient online services–enabled).

Appointment metric		Self-scheduled but staff could cancel	Staff-scheduled but patients could still self-cancel	*P* value^a^
**Appointment actions, n (%)**				
	Self-scheduled	13,454 (100)	0 (0)	<.001
	Staff-scheduled	0 (0)	117,656 (100)	<.001
	Self-cancelled	2166 (16.10)	3847 (3.27)	<.001
	Staff-cancelled	1855 (13.79)	36,278 (30.83)	<.001
	Total cancelled	4021 (29.89)	40,125 (34.10)	<.001
**Appointment outcomes, n (%)**				
	Finalized appointments (scheduled minus cancelled)	9433 (100)	77,531 (100)	N/A^b^
	Arrived to appointment	8897 (94.32)	73,941 (95.37)	<.001
	No-show	536 (5.68)	3590 (4.63)	<.001
**Appointment action efficiency**				
	Total appointment actions per finalized appointment (total count of the above 4 rows of self-scheduling and staff-scheduling and cancelling appointment actions divided by the total count of finalized appointments)	1.852	2.035	N/A
	Self-generated appointment actions per finalized appointment (total count of the above 2 rows of self-scheduled and self-cancelled appointment actions divided by the total count of finalized appointments)	1.656	0.050	N/A
	Staff-generated appointment actions per finalized appointment (total count of the above 2 rows of Mayo staff-scheduled and staff-cancelled appointment actions divided by the total count of finalized appointments)	0.197	1.985	N/A
**Appointment actions outside of standard appointment scheduler hours, n (%)**				
	Scheduling actions completed outside of normal business hours of Monday to Friday, 7 AM to 5 PM	3285 (24.42)	1659 (1.41)	<.001
	Scheduling actions completed on Saturday or Sunday	1149 (8.54)	769 (0.65)	<.001
	Scheduling actions completed on Monday to Friday outside of 7 AM to 5 PM	2136 (15.88)	890 (0.76)	<.001
**Appointment lead time**				
	Median lead time (days)	15	21	N/A
	Lead time over 84 days, n (%)	0 (0)	5778 (4.91)	<.001

^a^Null hypothesis (H0) tested: proportion of self-scheduled appointments equals staff-scheduled appointments.

^b^N/A: not applicable.

### Convenience of Scheduling

Approximately 24.4% (3285/13,454) of the mammogram self-scheduling activity was accomplished either on the weekend or on weekdays after usual staff scheduler hours (Table 2). This after-hours scheduling was done during the weekday for 15.9% (2136/13,454) of the appointment actions and on the weekend for 8.5% (1149/13,454) of the appointment actions. Approximately 75.5% (10,163/13,454) of the self-scheduling appointment actions were done via web and 24.5% (3291/13,454) of the appointment actions were done via mobile app.

### Scheduling Efficiency

**Figure 5:**
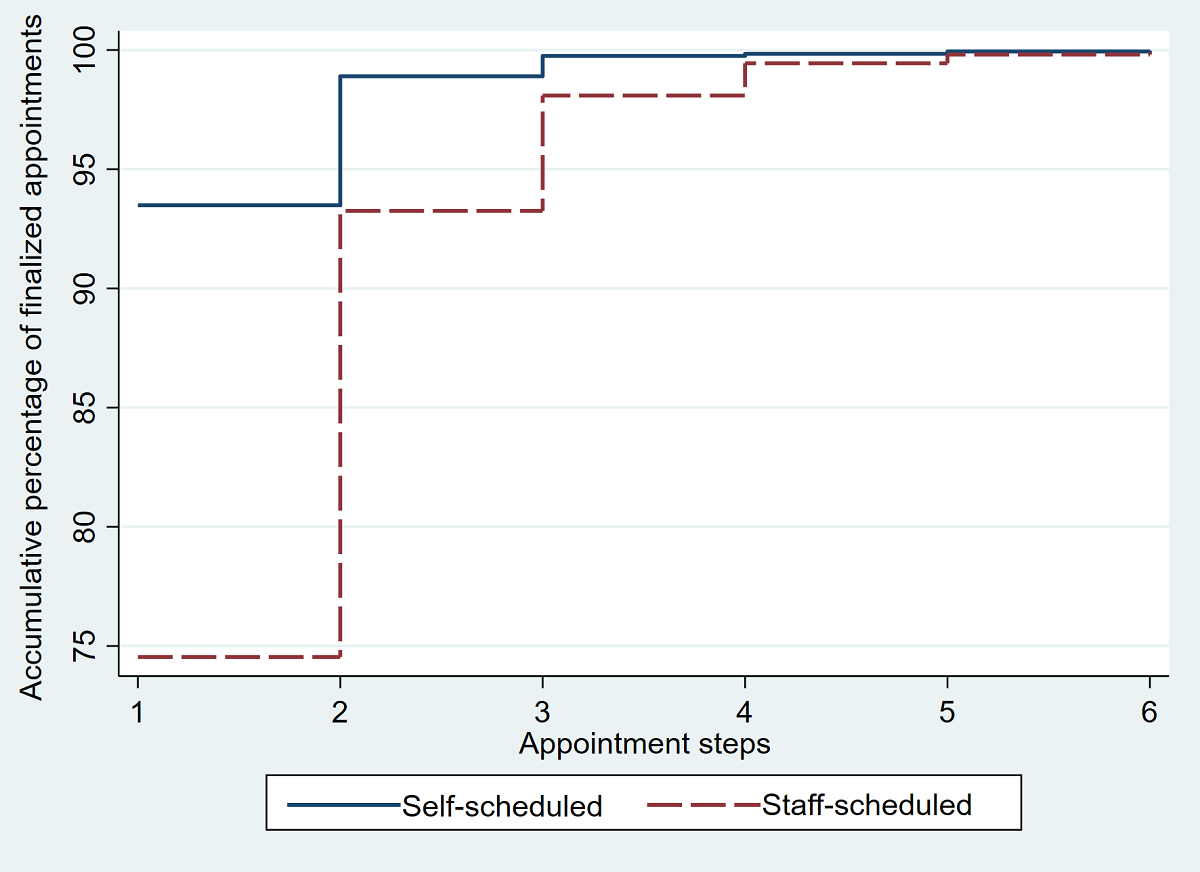
Comparison of accumulated percentage of exclusively self-scheduled finalized appointments to that of staff-scheduled finalized appointments by number of appointment steps completed. The graph shows that for each appointment step, the cumulative percentage of self-schedulers successfully completing the appointment process at that step was greater than that of those who used staff schedulers.

The average scheduling actions per finalized visit were similar between self-schedulers and staff schedulers (1.85 average self-scheduled appointment actions per finalized visit vs 2.04 for staff schedulers). There was not a major increase in scheduler work owing to self-scheduling. In fact, staff schedulers averaged only 0.197 appointment actions per finalized visit that had any self-scheduling. Thus, staff rework for patients attempting self-scheduling did not appear to be a major issue. Table 2 also shows that there was a smaller percentage of self-scheduled visits that were cancelled, and many of those were self-cancelled. For the exclusively self-scheduled visit, the appointment process was extremely efficient. Figure 5 shows that 93.5% (7553/8079) of the exclusively self-scheduled patients with a single finalized appointment were able to finalize that appointment in just 1 step (one and done). Thus, only 6.5% (526/8079) of the exclusively self-scheduled patients needed multiple appointment steps for a finalized appointment. However, 25.5% (18,035/70,839) of the staff-scheduled finalized appointments had multiple appointment steps; staff-scheduled finalized appointments took a single step in 74.5% (52,804/70,839) of the appointment cases. This resulted in an odds ratio of 4.90 (95% CI 4.48-5.37; *P*<.001) for multiple steps in scheduling when comparing staff scheduling to self-scheduling.

### Appointment Outcomes: No-shows

For the 12 months studied, there were bilateral screening mammograms scheduled for 93,901unique patients with patient online service access. Of the 131,110 mammograms scheduled, there were 44,146 cancellations, leaving 86,964 scheduled mammograms that were expected to be completed on the scheduled date. Of those appointments expecting to be completed, 95.3% (82,838/86,964) arrived for the visit for an overall no-show rate of 4.7% (4126/86,964) for those with patient online service access. Table 2 shows that the no-show rate for self-scheduled patients was 5.7% (536/9433) compared to 4.6% (3590/77,531) for the staff-scheduled patients. The unadjusted odds ratio of self-scheduled to staff-scheduled no-shows was 1.24 (95% CI 1.13-1.36; *P*<.001). Rosenbaum et al [16] found that patient age was a significant confounder in mammogram no-shows; therefore, we used a multivariable logistic regression model to adjust for age when examining differences in no-shows. In the age-adjusted model, the no-show rates were not significantly different; the age-adjusted odds ratio for self-scheduled to staff-scheduled no-shows was 1.09 (95% CI 0.99-1.20; *P*=.07). However, in a multivariable logistic regression model adjusting for race and ethnicity as well as age, we found a significant no-show odds ratio of self-scheduled to staff-scheduled of 1.12 (95% CI 1.02-1.23; *P*=.02).

### Appointment Outcomes: Lead Times

Self-scheduled patients were unable to make their mammogram appointment more than 12 weeks in advance. We found that 4.9% (5778/117,656) of staff-scheduled appointments were scheduled out further than 12 weeks.

### Sensitivity Analysis

The percentage of self-scheduled appointment actions is sensitive to the denominator used. Since self-scheduling requires patient online services, we used patients with patient online services as the denominator for our analysis. Portal engagement is not static in many practices. Mayo Clinic patient online services engagement increased from 33% to 62% during 2013 to 2018 [17], and 83.6% (93,901/112,367) of patients scheduling mammograms in our study had patient online services (Figure 3). When including all patients scheduling mammograms (patient online services–enabled or not), there were 151,165 mammograms scheduled over 12 months with a total of 208,521 scheduling and cancelling actions. For the entire cohort of patients with a scheduled mammogram, self-scheduling and self-cancelling patients performed 9.3% (19,467/208,521) of these actions with staff performing 90.7% (189,054/208,521) of these actions. As shown in Figure 4, the uptake of self-scheduling had increased substantially; therefore, the proportion of self-scheduled actions was also sensitive to the time frame examined. For the last 3 months of the study, the proportion of all self-scheduled mammogram actions (entire cohort of those patient online services–enabled or not) had increased to 12.6% (6109/48,447). The COVID-19 pandemic in spring 2020 resulted in an increase in mammogram cancellations both for self-scheduled and staff-scheduled mammograms. We separately analyzed the 6 pre- and post-COVID months (September 2019 through February 2020 and March 2020 through August 2020, respectively) for the average appointment actions per finalized visit. For self-scheduling, the 6 pre-COVID months had 0.186 staff-scheduling actions per finalized visit compared to 0.209 for the 6 post-COVID months. Thus, even with the COVID-19–associated cancellations, for appointments with any self-scheduling activity, there was only about 1 staff appointment action involved per 5 finalized appointments.

## Discussion

### Principal Findings

By 11 months, 21.1% (1991/9442) of the patients with self-scheduling access were engaged in self-scheduling their screening mammogram and 24.4% (3285/13,454) of the self-scheduling actions were outside of normal business hours for appointment scheduling. For 93.5% (7553/8079) of those who exclusively self-scheduled their screening mammograms, only 1 appointment step was used—that of a single step of choosing the date and time of the mammogram.

### Scheduler Work Implications

Patients performed a large number of scheduling actions, which otherwise would have been done by staff schedulers. There was very little staff-scheduler activity required for each finalized appointment in the self-scheduled group. Thus, there was not an unintended consequence of extra staff-scheduler work required to redo or “clean up” a self-scheduled appointment. We showed that the average self-scheduled finalized appointment involved only 0.197 staff actions compared to 2.04 staff actions on average required for a staff-scheduled finalized appointment. We did not measure the actual staff labor cost for each finalized appointment associated with self-scheduling and staff scheduling. However, with the average self-scheduled finalized visit using only 9.7% (0.197/2.04) of the staff appointment actions compared to a staff-scheduled appointment, there is likely a significant savings. Our findings suggest that the mammogram order generation and self-scheduling features will fit into the cost-effective multicomponent intervention framework for cancer screening identified by Mohan et al [18].

### Practice Implications

We did not identify major unintended consequences to the practice. No-shows were significantly greater for those in the self-scheduled group but were reduced to an odds ratio of 1.12 when adjusted for the patient age, race, and ethnicity differences noted in Table 1. Because automated bulk ordering of mammograms was part of the self-scheduling process, providers were freed up to do other activities besides ordering routine mammograms. As preventive services and other chronic care services take up an increasing amount of provider time, decreasing provider time for this activity is very important [8,9].

### Patient Implications

Patient self-scheduling is likely a benefit for many patients. We showed that many patients took advantage of the ability to self-schedule 24/7. With 24.5% (3285/13,454) of the self-scheduling occurring after business hours or on weekends and 24.5% (3291/13,454) of the self-scheduling occurring via mobile app, patients were using the anytime and anywhere capability of self-scheduling. Those who self-scheduled also were extremely efficient at doing so, with 93.5% (7553/8079) of their finalized appointments occurring after just 1 scheduling step. Mathioudakis et al [19] noted that women highly value time-efficient screening processes, and our data show the self-scheduling process to be efficient and convenient.

### Comparison With Other Studies

There appear to be few comparable studies for self-scheduled imaging. A review of web-based appointment scheduling by Zhao et al [20] focused on medical appointments rather than imaging appointments. Vendors such as Zocdoc or Lybrate offer web-based scheduling of medical appointments but not for imaging [13,21]. Compared to self-scheduled medical appointments, our first year uptake was similar. With a small sample size of 125, Zhang et al [22] found that 11% of patients had used a web-based appointment service for a primary health care center in Australia. We could not find a study like ours comparing no-show mammography appointment outcomes of self-scheduling to staff-scheduling. However, a study by Rosenbaum et al [16] showed a 6.99% no-show for mammography, which is somewhat higher than what we found with either self-scheduled or staff-scheduled mammogram appointments. In Rosenbaum et al’s study, it was noted that younger patients were more likely to no-show their imaging appointments. Given the lower ages in our self-scheduled group, perhaps age was a confounding factor that might explain the higher no-shows in the self-scheduled group. Consistent with Rosenbaum et al’s findings, when we adjusted for age in a multivariable logistic model, there was a nonstatistical difference in no-shows between self-scheduled and staff-scheduled mammogram appointments. However, further adjustment of our no-shows for race and ethnicity as well as age revealed a significant but small association of no-shows with self-scheduling.

The outcomes for self-scheduled mammograms show some interesting contrasts and similarities to outcomes for Mayo Clinic’s self-scheduled well-child visits [15]. Despite differences in patient populations (adult vs pediatric) and appointments scheduled (radiology procedure vs provider visits), there were similar scheduling efficiencies with 93.1% (712/765) of exclusively self-scheduled well-child visits being finalized with 1 appointment step compared to 1 appointment step needed for 93.5% (7553/8079) of the exclusively self-scheduled mammograms. A major difference was in the uptake of self-scheduling mammograms, which contrasted sharply with that of self-scheduled well-child visits. For the first year of implementation, the percentage of portal-registered unique patients using self-scheduling for mammograms was 15.3% (14,387/93,901) compared to 6.8% (1099/16,161) using well-child visit self-scheduling. An important difference from the well-child appointment process was that self-scheduling a mammogram was paired with a communication process that proactively alerted patients that a mammogram was due. From the user perspective, the electronic mammogram invitation not only notified them that the mammogram was due but also that they could self-schedule their mammogram from their mobile device or online and would not need to phone a scheduler. Although well-child appointments could be self-scheduled, they were not linked to an order that determined eligibility; no proactive well-child appointment due notices (scheduling invitations) were sent out. The pairing of alerting patients to schedule their mammogram appointment and allowing them to self-schedule in a “one stop” process may explain at least some of the two-fold differences in uptake between the mammogram self-scheduling and well-child self-scheduling. Since there were significant differences in population demographics, appointment types, and self-scheduling processes between the mammogram and well-child self-scheduled appointments, more work needs to be done to understand the differences and similarities in the outcomes.

### Limitations

Patients self-scheduling mammograms were 93.7% (13,474/14,382) White, and 83.6% (93,901/112,367) of all patients scheduling mammograms were registered with patient online services. Other populations could have different results. Even with our comparison limited to the 83.6% (93,901/112,367) of patients who had patient online service access, there were still significant differences in the ages of patients self-scheduling versus those using staff schedulers. The COVID-19 pandemic occurring in the last 6 months of this study limits some of our findings. However, our subgroup analysis into pre- and post-COVID time frames shows that the extra staff scheduler cancellations due to COVID was associated with only a small increase in average staff-scheduling activity in the self-scheduled group. No-show outcomes in imaging examinations are known to be influenced by a number of factors that we did not take into consideration. For example, in their review of over 3 million outpatient radiology visits, Mieloszyk et al [23] found significant associations of no-shows with patient income, commute distance, and daily snowfall. There is no uniform standard for mammogram screening; there are several somewhat differing recommendations from different specialty organizations and stakeholder groups [2,24,25]. Bitencourt et al [26] discuss some of the differences between breast cancer screening guidelines. Clinics that use different criteria for screening mammography may have different results.

We limited the mammogram self-scheduling feature to a 12-week window as mentioned above. This limits the conclusions about some of the scheduling efficiency. It is possible that the 12-week appointment window resulted in patients having more clarity on their future availability and some reschedules were avoided. Further, the inability to self-schedule more than 12 weeks in the future likely had an impact on the uptake of this feature. Since 4.9% (5778/117,656) of the staff-scheduled appointments were scheduled greater than 12 weeks out, there are likely patients who may have self-scheduled had they had the opportunity to schedule past the 12-week limit. We only examined mammograms that were scheduled. We did not look at the potential issues involved in the identification of individuals who met the criteria for generating a mammogram order. For example, if a mammogram had been recently done elsewhere, the patient might have been misidentified as being due for a mammogram. Further, patients who had changed their email or postal address and had not changed their address in their EHR might not have received their invitation for ordering a mammogram that was due. Our data only reflected those who had acted on screening mammogram orders. In this study, we did not evaluate the accuracy of the mammogram orders or if the patients had received their invitations.

### Future Research and Enhancements

Additional research will be needed to evaluate whether web and mobile mammogram self-scheduling will lead to a higher percentage of women receiving timely screening mammograms. A study by Gann et al [27] had an unexpected finding of a greater than 8% increase in mammogram utilization in practices with “active scheduling” compared to “passive scheduling.” “Active scheduling” was defined as patients engaged in scheduling their own mammogram, whereas “passive scheduling” was when the clinic actually made the appointment for the patient. Perhaps self-scheduling via web and mobile self-scheduling will be the internet equivalent of “active scheduling” and associated with increased mammogram utilization. Since there are patients who are having mammograms ordered and scheduled greater than 12 weeks in the future, a possible enhancement would be to expand that window of opportunity to self-schedule. A message to the patient noting that a mammogram would be due in 4-6 months and offering a wider window of future times to self-schedule could be an enhancement to evaluate.

### Conclusion

A large number of patients successfully self-scheduled their screening mammogram by using the web or mobile without staff-scheduler assistance. Self-scheduling actions were accomplished outside of normal staff-scheduling hours in 24.4% (3285/13,454) of the cases, and 93.5% (7553/8079) of exclusive self-scheduled mammogram appointments were done with just 1 appointment step (one and done). Self-scheduled screening mammograms were associated with more no-shows than staff-scheduled mammograms, with a small but significant odds ratio of 1.12 in a model adjusted for age, race, and ethnicity. There was no unintended consequence of an increase in staff-scheduler work because, on average, each finalized self-scheduled mammogram used less than one-tenth the staff-scheduler appointment actions compared to those completely staff-scheduled.

## Abbreviations

EHR: electronic health record
